# A Ca^2+^/Calmodulin-Interacting IQD Hub in Tartary Buckwheat: Genome-Wide *FtIQD* Analysis and Characterization of *FtIQD19*

**DOI:** 10.3390/plants15081212

**Published:** 2026-04-15

**Authors:** Guojun Chen, Chenyi Wu, Zhixing Zhao, Yuzhen Liang, Jingyi Wang, Zhenwang Li, Zhengyan Li, Xiule Yue

**Affiliations:** 1 Ministry of Education Key Laboratory of Cell Activities and Stress Adaptations, School of Life Sciences, Lanzhou University, Lanzhou 730000, China; 320220927891@lzu.edu.cn (G.C.); 320220928061@lzu.edu.cn (C.W.);; 2 The Institute of Animal Husbandry and Veterinary Medicine, Anhui Academy of Agricultural Sciences, Hefei 230031, China; lizhengyan1982@163.com

**Keywords:** *Fagopyrum tataricum*, IQ67-domain proteins, calmodulin, calcium signaling, abiotic stress, flavonoid, rutin

## Abstract

IQ67-domain (IQD) proteins are plant-specific calmodulin (CaM)/calmodulin-like (CML) targets implicated in the spatial organization of Ca^2+^ signaling, yet their roles in tartary buckwheat (*Fagopyrum tataricum*) remain largely unexplored. Here, we identified 24 *FtIQD* genes and classified them into six phylogenetic subfamilies. *FtIQD*s show uneven chromosomal distribution and mainly arise from segmental duplication under purifying selection. Promoter analysis revealed the enrichment of MYB-, light-, and ABA-related cis-elements. To link *FtIQDs* with rutin variation, we performed an *FtIQD*-focused association analysis using whole-genome resequencing data from altitude-stratified panels of up to 220 accessions. Under additive, dominant, and recessive models, multiple significant SNPs (*p* < 1 × 10^−5^) were detected near a subset of *FtIQD* loci, showing clear model- and environment-dependent patterns. Recurrent loci included *FtIQD22*, *FtIQD02*, *FtIQD16*, and *FtIQD19*. RNA-seq under PEG-induced drought stress, tissue expression patterns, pathway co-expression, and qRT–PCR further prioritized *FtIQD19*. FtIQD19–GFP showed predominant nuclear localization with additional filamentous/peripheral signals, and yeast two-hybrid assays identified FtCaM7.2 as the strongest interactor among representative CaMs. Structural modeling of the FtIQD19–FtCaM7.2 complex suggested testable residue-level interaction features. Collectively, this work provides a foundational FtIQD resource and highlights candidate Ca^2+^/CaM–IQD modules potentially associated with altitude-dependent rutin variation in tartary buckwheat.

## 1. Introduction

Tartary buckwheat (*Fagopyrum tataricum*) is a stress-resilient pseudocereal cultivated widely in marginal and highland environments [1]. In addition to its agronomic robustness, tartary buckwheat is valued for accumulating exceptionally high levels of flavonoids, particularly rutin and related flavonols (flavonol glycosides), which contribute to nutritional quality and are thought to enhance tolerance to oxidative stress and UV-B exposure [2]. Flavonoid content in tartary buckwheat varies substantially among genotypes and environments, and high-altitude cultivation often promotes flavonoid accumulation, consistent with increased UV-B and other altitude-associated cues [3,4]. Despite these ecological and nutritional advantages, the upstream signaling and regulatory nodes that connect environmental perception to flavonoid-related traits in tartary buckwheat remain incompletely understood.

Calcium (Ca^2+^) signaling is a central mechanism by which plants translate environmental inputs into cellular responses [5]. In tartary buckwheat, recent evidence further suggests that Ca^2+^ participates in stress-related physiological regulation and modulates antioxidant and phenylalanine/flavonoid-associated metabolism during seed germination [6]. Nevertheless, direct information on IQD-family genes or CaM/CML-related signaling components in tartary buckwheat remains limited. Diverse stresses, including drought, salinity, cold, and UV-B, rapidly elicit stimulus-specific Ca^2+^ signatures that are decoded by Ca^2+^ sensors and effectors, prominently calmodulins (CaMs) and calmodulin-like proteins (CMLs) [7]. CaM/CML proteins typically operate by binding target proteins in a Ca^2+^-dependent manner, thereby rewiring protein interactions, subcellular localization, and downstream transcriptional and metabolic programs [8]. Among the large repertoire of plant CaM-binding proteins, IQ67-domain (IQD) proteins form one of the largest plant-specific families and have been proposed to function as scaffold-like hubs that spatially organize Ca^2+^/CaM decoding in distinct cellular compartments [9,10].

Mechanistic studies in model plants have established that IQDs are multifunctional and often show pronounced subcellular patterning. A family-wide analysis in *Arabidopsis thaliana* revealed that many IQDs localize to microtubule (MT) arrays, plasma membrane subdomains, and nuclei and can recruit CaM to these sites, supporting a spatial signaling model for IQD function [11]. These properties make *IQD*s attractive candidates for linking Ca^2+^/CaM signaling with cytoskeletal dynamics, cell organization, and potentially broader transcriptional or metabolic responses under stress [10,11,12]. Representative members bind directly to MTs, promote MT stability, and influence cell-shape formation [13], and the DUF4005 region of ABS6/AtIQD16 functions as a bona fide MT-binding domain [14]. In crop species, growing evidence also links *IQD* genes to abiotic stress responses and tolerance. For example, potato IQD genes show differential expression under drought, extreme temperature, and CaCl_2_ treatments, whereas studies in apple and Chinese cabbage indicate that specific IQDs contribute to MT regulation and enhanced drought tolerance, respectively [15,16,17].

Despite these advances, the *IQD* family has not been systematically characterized in tartary buckwheat, and its possible contribution to flavonoid-related traits and stress-responsive metabolic regulation remains unresolved. Addressing this gap is particularly relevant because flavonoid accumulation in tartary buckwheat is shaped by complex regulatory circuits (light/UV-responsive signaling, hormone pathways, and transcription factor modules), and Ca^2+^/CaM-based decoding could provide an upstream layer that modulates these circuits in an environment-dependent manner [2,18,19,20]. Therefore, a systematic analysis of *FtIQD*s may provide candidate entry points for understanding stress adaptation and for developing genetic resources to improve nutritional and stress-resilience traits.

Here, we performed a comprehensive genome-wide identification and characterization of the *IQD* gene family in tartary buckwheat. We identified 24 *FtIQD* genes and analyzed their phylogeny, chromosomal distribution, duplication history, conserved motifs/domains, gene structures, and promoter *cis*-elements. To explore potential links between *FtIQD*s and rutin variation, we conducted a locus-focused association analysis using resequencing data from altitudinal populations and integrated the association signals with stress-responsive and tissue-specific expression profiles, pathway co-expression analysis, and qRT–PCR validation. Finally, we moved beyond family-level analysis to a preliminary characterization of FtIQD19: we determined its subcellular localization, validated interactions with representative CaMs, with FtCaM7.2 showing the strongest interaction, and used structural modeling to propose residue-level hypotheses for CaM recognition. Together, our results establish a resource for the *FtIQD* family, nominate prioritized candidates for functional validation, and provide a testable framework linking Ca^2+^/CaM decoding to environment-dependent rutin variation in tartary buckwheat.

## 2. Results

### 2.1. Identification and Physicochemical Features of FtIQD Genes

Using the intersection of results from BLASTP searches and HMMER scans for the IQ67 domain, we identified 24 IQ67-domain (*IQD*) genes in the tartary buckwheat genome and designated them *FtIQD01–FtIQD24* according to their chromosomal positions (Table 1). Genomic sequence lengths ranged from 623 bp (*FtIQD23*) to 3829 bp (*FtIQD07*), and CDS lengths ranged from 531 bp (*FtIQD23*) to 1641 bp (*FtIQD19*) (Table 1). The predicted FtIQD proteins varied from 176 aa (FtIQD23) to 546 aa (FtIQD19), with molecular weights of 19.9–61.7 kDa. All FtIQD proteins were predicted to be basic, with theoretical pI values between 9.5 and 10.6 (Table 1).

Because IQD proteins typically act as Ca^2+^ signal decoders through calmodulin (CaM)/CaM-like (CML) recruitment, we further screened FtIQDs for potential CaM-binding segments. Putative CaM-binding regions were detected in all FtIQD proteins (Table S1), providing candidates for downstream interaction validation and functional inference [9].

### 2.2. Phylogeny and Genomic Organization of FtIQDs

**Figure 1:**
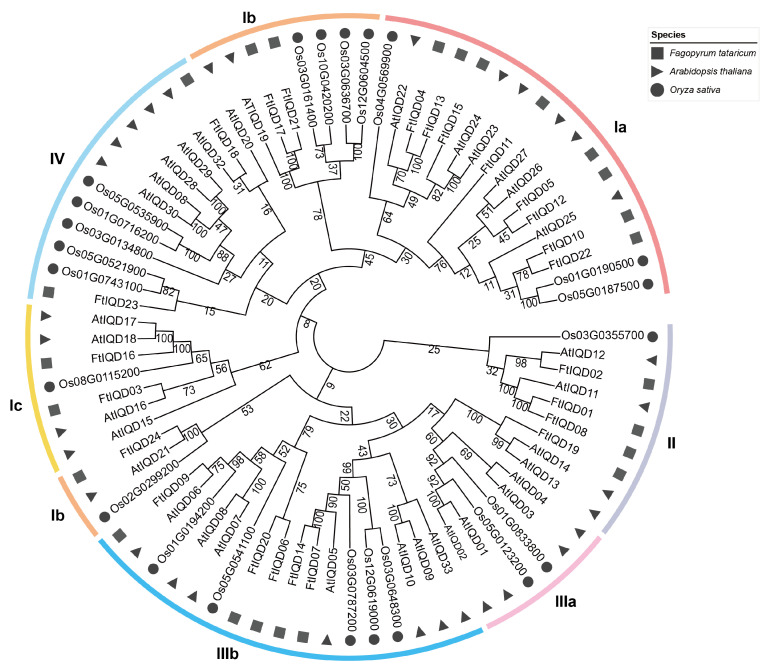
Phylogenetic relationships of IQD proteins from *Fagopyrum tataricum*, *Arabidopsis thaliana*, and *Oryza sativa*. The neighbor-joining tree is constructed based on full-length IQD protein sequences with 1000 bootstrap replicates. Bootstrap support values (%) are shown on the branches. *FtIQD* subfamilies are indicated by colored strips, and species are distinguished by different symbols.

To place FtIQDs in an evolutionary framework, we constructed a neighbor-joining phylogeny based on IQD proteins from *Arabidopsis thaliana*, *Oryza sativa*, and *Fagopyrum tataricum* (Figure 1). Based on the established Arabidopsis IQD subfamily system, phylogenetic analysis classified the FtIQDs into six subfamilies (Ia, Ib, Ic, II, IIIb, and IV). Notably, no FtIQD member was placed within the IIIa clade (Figure 1). FtIQD23 (subfamily IV) grouped closely with two rice orthologs (Os05t0521900 and Os01t0743100), while most FtIQDs showed closer affinity to Arabidopsis members, consistent with the shared dicot lineage.

**Figure 2:**
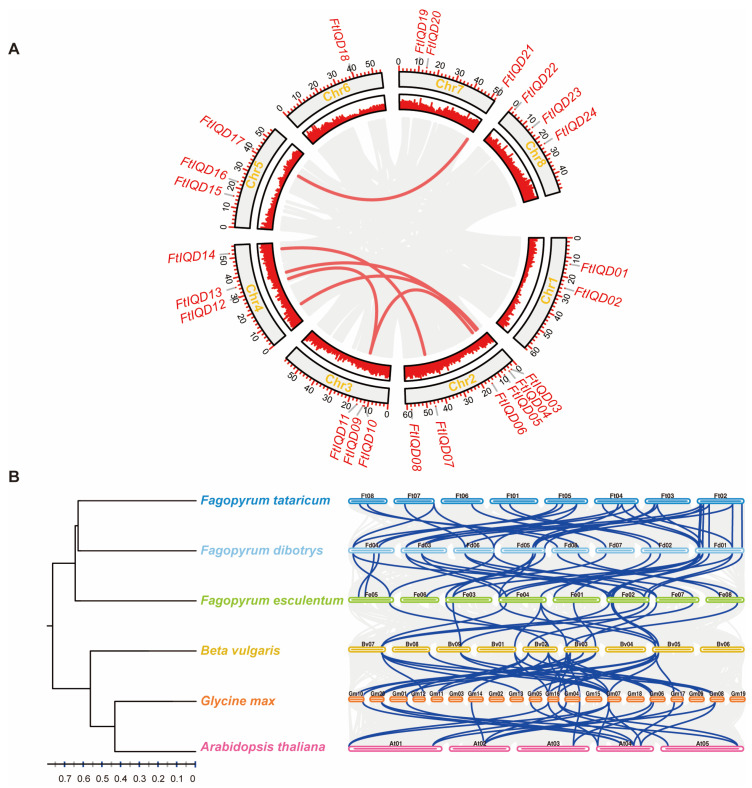
Synteny analysis of *FtIQD* genes: (**A**) Intraspecific collinearity of *FtIQD* genes in tartary buckwheat. The outer circle represents the eight chromosomes, and *FtIQD* loci are marked on the corresponding chromosomes. The inner circle displays the gene density. Collinear *FtIQD* gene pairs are connected by red lines. (**B**) Interspecific synteny of *IQD* genes among six plant species. Each horizontal block represents a chromosome. Grey lines indicate collinear genomic blocks, and blue lines indicate syntenic gene pairs corresponding to *FtIQD*s.

The 24 *FtIQD*s were distributed across all eight chromosomes (Figure 2A). Chromosome 2 contained the largest number (six genes), chromosome 6 contained only one gene, and the remaining chromosomes each harbored three genes. *FtIQD*s were unevenly positioned along chromosomes and tended to locate toward chromosomal arms rather than near centromeric regions. Intra-species collinearity analysis identified five duplicated gene pairs (ten genes) within collinear blocks. These pairs were consistent with segmental duplication, as no duplicated pairs were adjacent on the same chromosome (Figure 2A). Ka/Ks ratios for duplicated pairs ranged from 0.138 to 0.359 (Table S2), indicating predominant purifying selection after duplication. Notably, *FtIQD19* and *FtIQD22* lacked detectable syntenic homologs in the surveyed *Fagopyrum* genomes (Figure 2B). These results reflect the current synteny-based detection given the available assemblies and annotations.

### 2.3. Structural Features, Conserved Motifs, and Cis-Regulatory Elements

**Figure 3:**
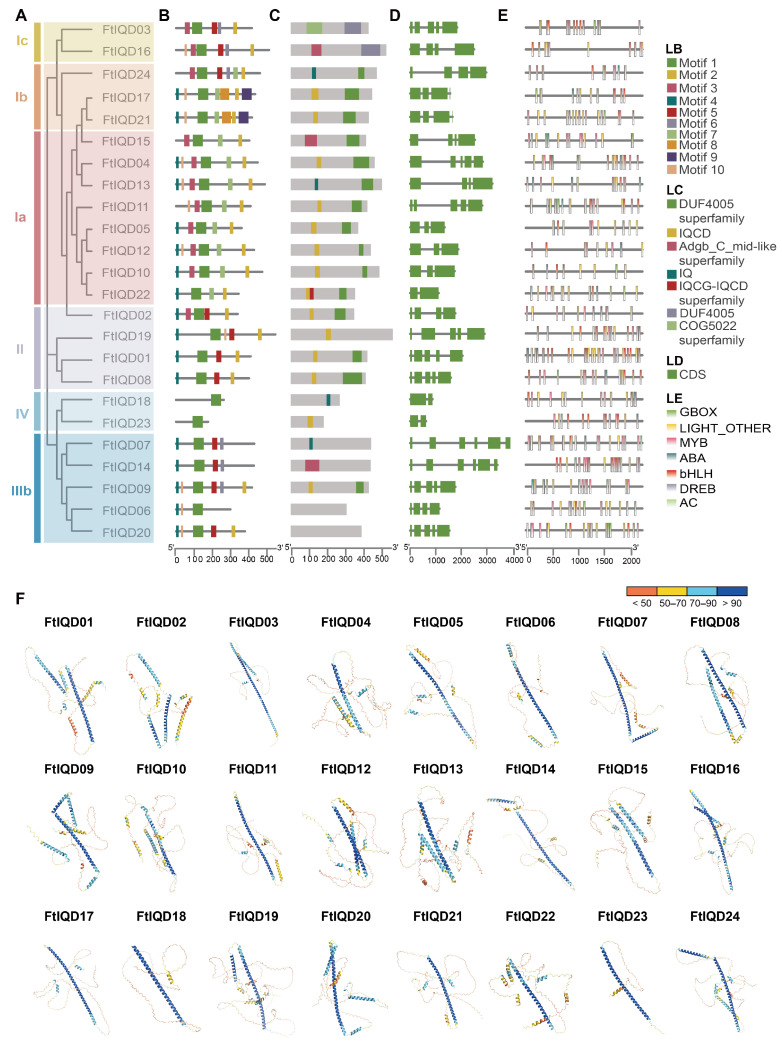
Sequence features, gene structures, cis-elements, and predicted protein structures of FtIQD members: (**A**) Phylogenetic tree of FtIQD proteins constructed using the neighbor-joining method in MEGA 7.0 with 1000 bootstrap replicates. Subfamily classification is assigned by reference to the Arabidopsis IQD clades. (**B**) Conserved motifs identified in FtIQD proteins by MEME (v5.5.9). Each motif is represented by a colored box, and identical motif numbers indicate the same motif across proteins (legend: LB). (**C**) Conserved domain architecture of FtIQD proteins (legend: LC). (**D**) Exon–intron structures of *FtIQD* genes. Green boxes indicate exons, and black lines indicate introns (legend: LD). (**E**) Distribution of representative cis-acting elements in 2-kb promoter regions of *FtIQD* genes (legend: LE). (**F**) AlphaFold-predicted structures of FtIQD proteins colored by per-residue confidence (pLDDT): >90 (dark blue), 70–90 (light blue), 50–70 (yellow), and <50 (orange).

MEME analysis identified ten conserved motifs in FtIQD proteins (Figure 3A,B). Motif 1 was present in all FtIQDs, consistent with it forming part of the conserved IQD core. Motif 2 was absent from FtIQD06 (IIIb), FtIQD07 (IIIb), FtIQD14 (IIIb), FtIQD18 (IV), and FtIQD23 (IV), while several motifs showed subfamily-preferential distributions, suggesting functional diversification after subfamily radiation [21]. Conserved domain annotation confirmed the presence of IQ motif-related regions (IQ and IQCD), with partial overlap between motif 1 and the annotated IQD core (Figure 3C). Analysis of *FtIQD* coding sequences (CDSs) showed broadly conserved organizations within subfamilies (Figure 3D).

To infer potential upstream regulatory inputs, we surveyed 2 kb promoter regions using PlantCARE and categorized cis-elements into seven functional groups (Figure 3E). MYB-related motifs (29.6%) and light-responsive elements (LIGHT_OTHER, 27.2%) were most abundant, followed by bHLH-binding motifs (17.0%), ABA-responsive elements (14.2%), and G-box motifs (11.1%). In contrast, DREB- and AC-type elements were rare (<1% each). At the gene level, promoters of several *FtIQD*s showed comparatively high densities of ABA- and/or light-responsive elements. For example, *FtIQD20* contained multiple ABA-related motifs and G-box/light elements, whereas *FtIQD14* displayed a MYB/bHLH-dominant cis-element composition.

AlphaFold predictions indicated that FtIQD proteins were predominantly alpha-helical, consistent with the helical propensity of the IQ67 region (Figure 3F). Per-residue confidence (pLDDT) was generally higher across the helical core and lower in terminal/linker segments. Together with motif/domain conservation, these features are compatible with a scaffold-like role for FtIQDs in assembling protein complexes, including CaM-associated modules [22].

### 2.4. FtIQD-Focused Association Analysis for Rutin Content

**Figure 4:**
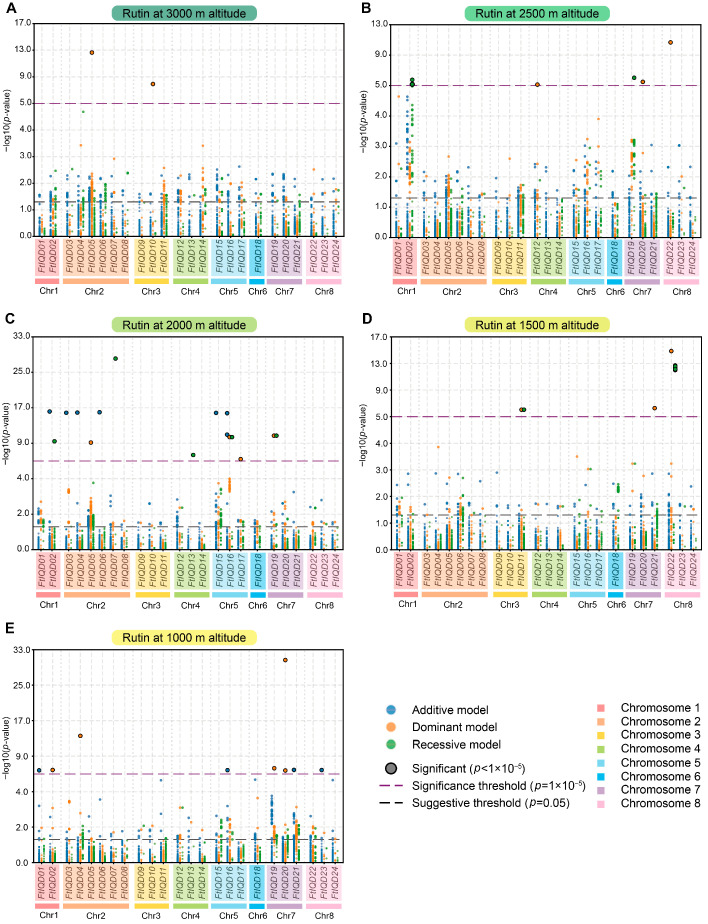
*FtIQD*-focused association analysis of rutin content across altitudinal groups. Association results for rutin content are shown for 3000 m (**A**), 2500 m (**B**), 2000 m (**C**), 1500 m (**D**), and 1000 m (**E**). The x-axis shows *FtIQD* loci ordered by chromosomal position from chromosomes 1 to 8, and the y-axis shows association significance as −log_10_(*p*). For each locus, the additive, dominant, and recessive models are plotted together. Column colors indicate chromosomes. The black dashed line marks the nominal threshold (*p* = 0.05; −log_10_(*p*) = 1.30), whereas the purple dashed line marks the stringent threshold (*p* = 1 × 10^−5^; −log_10_(*p*) = 5). Bold dots with black borders denote the most significant SNPs in each panel.

To evaluate the relationship between *FtIQD* loci and rutin variation, we performed an *FtIQD*-focused association analysis using whole-genome resequencing data from expanded altitude-stratified panels of up to 220 tartary buckwheat accessions. Because rutin is the major and most biologically relevant flavonoid in tartary buckwheat, the analysis was focused specifically on this trait. SNPs located within the *FtIQD* gene bodies and flanking regions (as defined in Methods) were tested under additive, dominant, and recessive genetic models across five altitudinal environments (1000, 1500, 2000, 2500, and 3000 m). Significant sites exceeding a stringent threshold (*p* < 1 × 10^−5^; −log10(*p*) > 5) are shown in Figure 4. Detailed information on the significant SNPs is provided in Table S3.

Across the three models and five environments, significant SNPs were detected in or near a limited subset of *FtIQD* loci, but their distribution varied substantially among genetic models and altitudes, indicating a pronounced model-dependent and environment-dependent genetic architecture for rutin accumulation. Several loci emerged repeatedly across models and/or environments, including *FtIQD22*, *FtIQD02*, *FtIQD16*, and *FtIQD19*. *FtIQD19* was among the recurrent loci subsequently retained for downstream evaluation. *FtIQD20* exhibited a particularly strong dominant-model signal at 1000 m, and a prominent recessive-model signal was observed for *FtIQD07* at 2000 m. Other loci, including *FtIQD04*, *FtIQD05*, *FtIQD10*, and *FtIQD21*, displayed significant but more model- or environment-specific signals.

Overall, the multi-model association analysis highlighted a focused set of *FtIQD* loci associated with rutin variation in tartary buckwheat and provided an association-based line of evidence for downstream candidate prioritization. To maintain focus on rutin, the corresponding association results for quercetin are provided in Supplementary Figure S1 and Table S4.

### 2.5. Expression Patterns and Correlation with Flavonoid Biosynthesis

**Figure 5:**
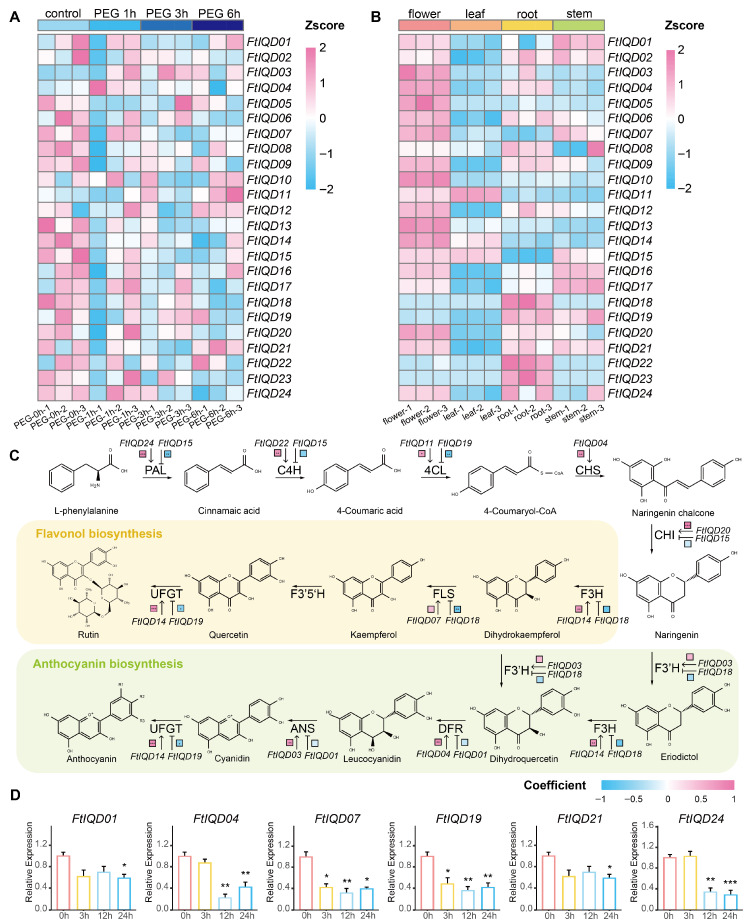
Expression profiles and correlation analysis of *FtIQD* genes: (**A**) Expression patterns of *FtIQD*s under PEG-induced drought stress based on a publicly available RNA-seq dataset (PRJCA003569). “PEG 0 h”, “PEG 1 h”, “PEG 3 h”, and “PEG 6 h” represent control and 20% PEG6000-treated samples collected at 0, 1, 3, and 6 h, respectively. Values are shown as z-score–normalized log_2_(TPM + 1). (**B**) Tissue-specific expression patterns of *FtIQD*s in leaves, roots, stems, and flowers, shown as z-score-normalized transcript levels. (**C**) Spearman correlation between *FtIQD* expression levels and enzymes in the flavonol/anthocyanin biosynthesis pathway. For each enzyme, only the strongest positive and strongest negative correlations are shown. Colors indicate Spearman’s correlation coefficients, and asterisks denote significance (* *p* < 0.05; ** *p* < 0.01; *** *p* < 0.001). Abbreviations: PAL, phenylalanine ammonia-lyase; C4H, cinnamic acid 4-hydroxylase; 4CL, 4-coumarate:CoA ligase; CHS, chalcone synthase; CHI, chalcone isomerase; F3H, flavanone 3β-hydroxylase; F3′H, flavonoid 3′-hydroxylase; F3′5′H, flavonoid 3′,5′-hydroxylase; FLS, flavonol synthase; DFR, dihydroflavonol 4-reductase; ANS, anthocyanidin synthase; UFGT, flavonoid 3-O-glucosyltransferase. (**D**) qRT–PCR validation of *FtIQD01*, *FtIQD04*, *FtIQD07*, *FtIQD19*, *FtIQD21*, and *FtIQD24* in 11-day-old tartary buckwheat seedlings under 20% PEG6000 treatment for 0, 3, 12, and 24 h. Expression levels are normalized to the *FtH3* gene and calculated using the 2^−ΔΔCt^ method relative to 0 h. Error bars indicate SD of 3–4 biological replicates. Asterisks indicate significance versus 0 h (* *p* < 0.05; ** *p* < 0.01; *** *p* < 0.001; Student’s *t*-test).

We next examined *FtIQD* expression patterns using publicly available RNA-seq datasets for PEG6000-induced drought stress (PRJCA003569), with samples collected at 0, 1, 3, and 6 h. Genes with extremely low read abundance across samples (as defined in Methods) were excluded from differential-expression interpretation. For the remaining genes, PEG treatment induced overall modest changes (|log_2_FC| < 1), but distinct expression trends were still evident when visualized as log_2_(TPM + 1) values with row-wise z-score normalization (Figure 5A). Fifteen genes showed a downward trend across the sampled time course (including *FtIQD01*, *FtIQD19*, and *FtIQD24*), whereas four genes (*FtIQD04*, *FtIQD10*, *FtIQD11*, and *FtIQD12*) showed a relative upward trend.

Tissue-specific expression analysis revealed clear organ partitioning (Figure 5B). Ten genes, particularly *FtIQD03* and *FtIQD05*, showed the highest expression in flowers, while *FtIQD11*, *FtIQD14*, and *FtIQD15* were enriched in leaves. In contrast, *FtIQD18*, *FtIQD22*, and *FtIQD23* were strongly expressed in roots, and multiple genes displayed elevated expression in stems.

To evaluate potential connections between *FtIQDs* and genes involved in flavonoid/anthocyanin biosynthesis, we calculated Spearman correlations between FtIQD expression and key pathway genes (Figure S2). Among the terminal pathway genes, *FtIQD14* showed the strongest positive correlation with UFGT (flavonoid 3-O-glucosyltransferase), whereas *FtIQD19* showed the strongest negative correlation with UFGT (Figure 5C). Overall, *FtIQD15*, *FtIQD18*, and *FtIQD23* exhibited the largest number of strong correlations, followed by *FtIQD01*, *FtIQD03*, *FtIQD04*, *FtIQD14*, and *FtIQD19* (Figure 5C; Figure S2). As *FtIQD23* showed correlation coefficients and significance patterns identical to those of FtIQD18, it was omitted from Figure 5C for clarity.

Based on a combination of drought-responsive expression trends in the RNA-seq data and repeated *FtIQD*-focused association signals for rutin content, we selected six genes (*FtIQD01*, *FtIQD04*, *FtIQD07*, *FtIQD19*, *FtIQD21*, and *FtIQD24*) for qRT-PCR validation in 11-day-old tartary buckwheat seedlings under a 20% PEG6000 time course. In contrast to the RNA-seq dataset from BioProject PRJCA003569, this validation experiment included additional sampling points at 12 h and 24 h to provide extended temporal resolution. All six genes were significantly downregulated over time, with *FtIQD04*, *FtIQD07*, *FtIQD19*, and *FtIQD24* showing the most pronounced decreases (Figure 5D; Table S5) [23,24]. We then combined *FtIQD*-focused association results with qRT–PCR-based stress-response profiles to prioritize targets for downstream functional validation [25]. Collectively, the convergence of association signals, stress-responsive expression, and pathway-linked co-expression supported *FtIQD19* as a prioritized candidate for further experimental investigation.

### 2.6. Experimental Validation and Model-Based Interpretation for FtIQD19

**Figure 6:**
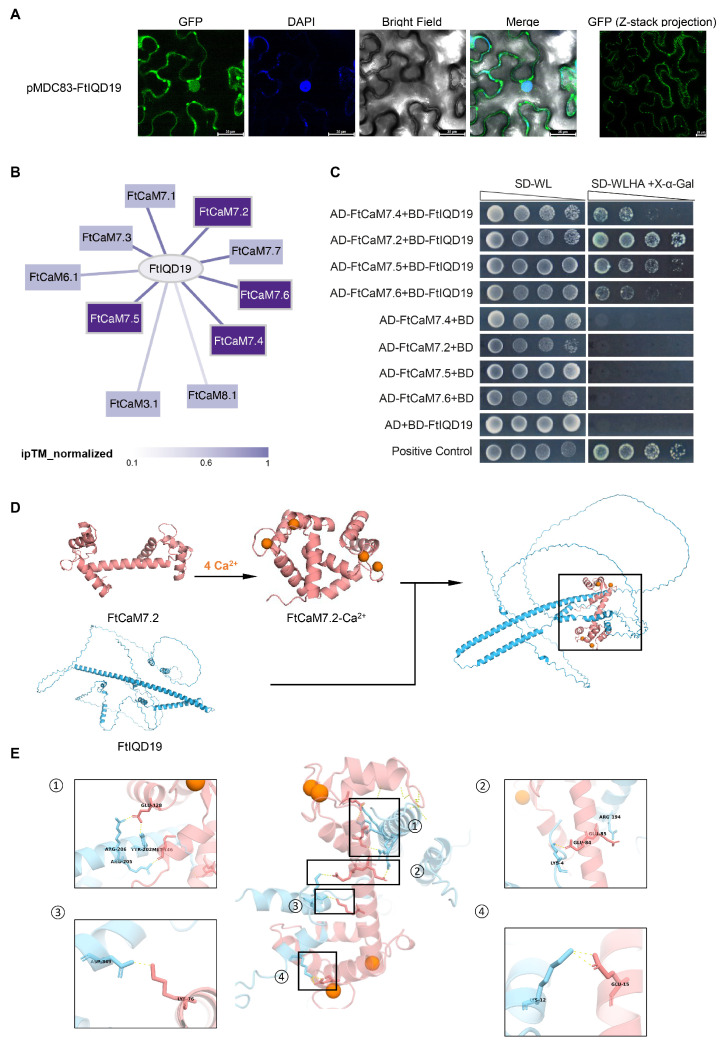
Subcellular localization and CaM interaction analysis of FtIQD19: (**A**) Subcellular localization of FtIQD19 in tobacco epidermal cells. GFP, DAPI, bright-field, and merged images were captured after transient expression of pMDC83–FtIQD19. A Z-stack projection of the GFP channel is shown in the last panel. Scale bars represent 35 µm (GFP, DAPI, bright-field, and merged images) and 20 µm (Z-stack projection). (**B**) Predicted interaction network between FtIQD19 and FtCaM candidates based on AlphaFold Multimer confidence scores (ipTM_normalized). Nodes represent FtIQD19 (center) and predicted FtCaM partners; node colors indicate whether the partner was selected for experimental testing. Edge transparency and length are inversely scaled to ipTM_normalized, such that darker and shorter edges represent stronger predicted interactions. (**C**) Yeast two-hybrid validation of FtIQD19 interactions with representative FtCaM proteins. The bait construct pGBKT7–FtIQD19 and prey constructs (FtCaM7.2, FtCaM7.4, FtCaM7.5, and FtCaM7.6) are co-transformed into yeast strain Y2HGold and tested on SD/−WL and SD/−WLHA supplemented with X-α-Gal. (**D**,**E**) Structural model of the FtIQD19–FtCaM7.2 complex predicted by AlphaFold Multimer. (**D**) Overall structure of FtCaM7.2, Ca^2+^-bound FtCaM7.2, FtIQD19, and the docked complex; Ca^2+^ ions are shown as orange spheres. (**E**) Key interface residues are shown in stick representation, with boxed insets highlighting local interactions.

To gain insight into the cellular context of FtIQD19, we transiently expressed an FtIQD19–GFP fusion in *Nicotiana benthamiana* leaves and examined fluorescence by confocal microscopy. FtIQD19–GFP signals overlapped with DAPI staining, indicating predominant nuclear localization (Figure 6A). In addition to the nuclear signal, z-stack imaging revealed filamentous fluorescence extending toward the cell periphery, consistent with a potential association with cytoskeletal structures or membrane-proximal compartments [10,11].

To explore potential CaM partners, we identified 10 putative CaM homologs in tartary buckwheat (Table S6) and predicted FtIQD19–CaM interactions using AlphaFold Multimer. The predicted confidence scores (ipTM_normalized) suggested relatively stronger predicted interactions with CaM7-like proteins (normalized score of 1 for FtCaM7.1 and FtCaM7.2) compared to CaM6.1 (0.5833) and CaM3.1 (0.4583), while interactions with CaM8 members were notably weaker (normalized scores ranging from 0.1667 to 0.3333) (Figure 6B). We then performed yeast two-hybrid assays to validate representative CaM interactions. Co-transformants grew on SD/−Trp/−Leu (SD/−WL; transformation control), while empty-vector controls failed to grow on stringent interaction medium (SD/−Trp/−Leu/−His/−Ade (SD/−WLHA) + X-α-Gal), supporting assay validity (Figure 6C). Among the tested pairs, FtCaM7.2 showed robust growth and blue coloration across dilution gradients, indicating a strong interaction with FtIQD19.

Finally, to elucidate the structural basis of the FtIQD19–FtCaM7.2 interaction, we modeled the Ca^2+^-bound complex using AlphaFold Multimer (Figure 6D,E). The predicted structure reveals a canonical calmodulin fold for FtCaM7.2 and a putative interface involving both electrostatic contacts and a prominent hydrophobic insertion. An aromatic anchor-and-pocket interaction was predicted, in which FtIQD19-Tyr202 inserts into a hydrophobic pocket within the C-terminal lobe of FtCaM7.2 [26]. This insertion was further secured by an adjacent salt bridge between FtIQD19-Arg206 and FtCaM7.2-Glu128. Additional electrostatic contacts included a flanking interaction in which FtIQD19-Lys4 and Arg194 bracket the acidic Glu84/85 cluster of FtCaM7.2, as well as a direct salt bridge between FtIQD19-Asp349 and FtCaM7.2-Lys76 and an intramolecular salt bridge between FtIQD19-Lys12 and FtIQD19-Glu15. Together, these model-based features provide testable hypotheses for future mutational and biochemical validation [27].

## 3. Discussion

Plants translate fluctuating environmental cues into adaptive transcriptional and metabolic outputs through layered signaling networks. Ca^2+^ transients are among the earliest stress signals, and calmodulins (CaMs) and calmodulin-like proteins (CMLs) decode these signatures by binding downstream targets. IQ67-domain (IQD) proteins represent a large plant-specific class of CaM/CML targets and are increasingly viewed as scaffold-like hubs that position Ca^2+^/CaM decoding within distinct subcellular contexts, thereby linking Ca^2+^ signaling to cytoskeleton dynamics and nuclear outputs [10]. Tartary buckwheat (*Fagopyrum tataricum*) is particularly suitable for exploring signaling–metabolism connections because it combines strong stress resilience with high flavonoid accumulation, especially rutin. Flavonoid levels vary substantially across genotypes and environments, and altitude-associated cues (e.g., UV-B intensity and temperature fluctuations) can reshape protective flavonoid allocation [2,18,19]. In this context, our study establishes a foundational *FtIQD* family resource and integrates genetic signals across environments with expression-based prioritization and initial mechanistic evidence for *FtIQD19*, thereby generating testable hypotheses for how Ca^2+^/CaM-associated scaffolds may contribute to environment-dependent rutin variation.

### 3.1. Evolutionary Features of the FtIQD Family and Implications for Functional Diversification

We identified 24 *FtIQD* genes in tartary buckwheat and classified them into six Arabidopsis-defined subfamilies (Ia, Ib, Ic, II, IIIb, and IV), with no *FtIQD* member placed within the IIIa clade. This pattern may reflect lineage-specific loss or substantial divergence after species separation rather than strict conservation of subfamily composition across taxa. It also highlights a methodological caveat: subfamily boundaries defined in a model species may not transfer cleanly to non-model genomes when grouping is driven mainly by overall sequence similarity. Accordingly, the combined use of phylogeny, motif/domain composition, gene structure, and genome-context evidence (e.g., synteny and duplication history) provides a more conservative framework for describing *FtIQD* diversification.

Within tartary buckwheat, duplicated *FtIQD* pairs are consistent with segmental duplication and show Ka/Ks ratios well below 1, indicating predominant purifying selection. This pattern supports the conservation of core *IQD* functions while allowing diversification through regulatory rewiring and shifts in interaction specificity. Comparative synteny revealed strong conservation within the *Fagopyrum genus* but fewer orthologous relationships in more distant dicots, as expected from genome rearrangements and differential gene retention. Notably, *FtIQD19* and *FtIQD22* lacked detectable syntenic homologs in the surveyed *Fagopyrum* genomes. A parsimonious explanation is lineage-specific gain/loss or rapid sequence divergence that weakens synteny-based detection; alternatively, differences in genome assembly or annotation among reference genomes may contribute to the apparent absence. Either scenario is compatible with the possibility that a subset of *FtIQD*s may represent lineage-biased innovations relevant to ecological adaptation.

### 3.2. IQDs as Spatial Interfaces Between Ca^2+^/CaM Signaling, Microtubules, and Stress Responses

Mechanistic work in *Arabidopsis* provides strong evidence that IQDs can interface with microtubules (MTs) and influence MT organization. IQD5 binds directly to MTs, promotes MT stability, and affects subsequent cell-shape formation [28]. In addition, the DUF4005 region of ABS6/AtIQD16 functions as a bona fide MT-binding domain, as demonstrated by in vitro MT co-sedimentation and in vivo MT decoration [14]. Together with family-wide localization diversity (nucleus, MT arrays, and membrane-associated compartments), these findings support a spatial signaling model in which IQDs recruit CaM/CML to defined cellular sites and thereby shape local signaling outputs [10].

Beyond *Arabidopsis*, accumulating evidence links *IQD*s to abiotic stress tolerance. Cotton *IQD* genes respond to drought, salt, and cold and have been proposed to participate in stress-associated signaling [22,29]. In *Nicotiana benthamiana*, ectopic expression of *BrIQD35* enhances drought tolerance, and the protein displays CaM-isoform-dependent interaction behavior, suggesting that partner preference may tune signaling specificity under different Ca^2+^ signatures [17]. In apple, selected *IQD*s associate with MTs and were proposed to contribute to MT stability under cold stress [16]. Collectively, these studies indicate that *IQD*s are well positioned to couple Ca^2+^ decoding to cellular architecture and stress acclimation, providing a relevant framework for interpreting *FtIQD* candidates in tartary buckwheat.

### 3.3. Integrating Association and Expression Evidence Highlights Candidate FtIQDs for Rutin-Related Traits

A notable feature of this study is the use of additive, dominant, and recessive models to examine *FtIQD*-focused associations with rutin content across multiple altitudinal groups. Because the allelic mode of action at candidate *FtIQD* loci was not known a priori, the three models were treated as complementary analytical frameworks rather than competing alternatives [30,31]. The detection of significant loci under all three models suggests that rutin-related variation within *FtIQD* intervals is unlikely to be explained by a single predominant inheritance pattern but instead reflects a mixture of allelic effects. The strong heterogeneity of association signals among altitude groups further indicates that the genetic architecture underlying rutin accumulation is environmentally contingent. This interpretation is biologically plausible because altitude is associated with coordinated shifts in UV exposure, temperature fluctuation, oxidative stress, and other ecological factors that influence flavonoid metabolism [2,3,20]. At the same time, altitude stratification changes the statistical context of association testing, including sample composition, allele frequency, and genotype-class representation. The altitude dependence observed here, therefore, likely reflects both biological context specificity and differences in detection power, supporting a role for genotype-by-environment interactions in shaping rutin accumulation [3,23,32].

Recent tartary buckwheat studies have linked rutin variation to biosynthetic enzymes and regulators, including FtS1Fa1- and UFGT-related modules [19,33,34]. In contrast, the present study highlights a set of upstream Ca^2+^/CaM-associated *IQD* candidate loci. Among the loci emerging from the *FtIQD*-focused analysis, *FtIQD22*, *FtIQD02*, *FtIQD16*, *FtIQD19*, and *FtIQD20* represent the most recurrent association candidates. *FtIQD22* contained the largest number of significant SNPs, whereas *FtIQD20* harbored one of the strongest model-specific association peaks. *FtIQD02* and *FtIQD16* were supported by multiple significant variants in specific altitude groups, and *FtIQD07* also showed a prominent model-specific peak at 2000 m. Importantly, *FtIQD19* recurred across models and/or environments rather than appearing as a single context-specific hit, which made it particularly suitable for downstream evaluation when statistical and molecular evidence were considered together.

Although several loci exceeded the predefined significance threshold in the *FtIQD*-focused analysis, these signals should still be interpreted as candidate-prioritization evidence rather than direct proof of causality. Rutin accumulation is likely a complex quantitative trait controlled by multiple loci of modest to moderate effect, and even within a focused candidate-gene framework, association alone cannot establish the mechanism [19,34,35]. For this reason, we used expression analyses as an independent axis for candidate refinement. Tissue-partitioned expression indicates that subsets of *FtIQD*s likely operate in organ-specific signaling contexts, and co-expression analysis implicated multiple *FtIQD*s as being associated with enzymes in the flavonol/anthocyanin pathway. This is biologically relevant because buckwheat flavonoid accumulation is controlled by multi-layer regulatory modules (e.g., MYB/bHLH and light/UV-responsive regulators) and by key glycosyltransferase steps required for rutin biosynthesis [34,36]. Thus, convergence of (i) recurrent association signals, (ii) stress-responsive expression, (iii) tissue distribution, and (iv) co-expression with pathway genes provides a rational basis for prioritizing FtIQDs as candidate regulators of rutin-related traits.

### 3.4. FtIQD19 Engages a CaM Partner and Shows Nuclear-Plus-Filamentous Localization: Mechanistic Implications

A key advance of this study is the combination of localization and CaM interaction assays for FtIQD19. FtIQD19–GFP exhibited predominant nuclear fluorescence together with filamentous/peripheral signals. This dual pattern is notable because *Arabidopsis* IQDs display diverse localization across nuclei, MT arrays, and membrane-associated sites, and such spatial partitioning has been linked to IQD-dependent CaM recruitment [10]. While our current data do not resolve the identity of the filamentous structures, the pattern is consistent with a spatial organizer role and motivates targeted co-localization with MT and membrane markers under control and stress conditions.

We further provided molecular evidence that FtIQD19 can engage CaM proteins. Guided by AlphaFold Multimer predictions, we prioritized CaM7 candidates and performed yeast two-hybrid assays with representative FtCaMs (FtCaM7.2, FtCaM7.4, FtCaM7.5, and FtCaM7.6), which identified FtCaM7.2 as the strongest interactor. This result is consistent with the idea that IQDs can show partner preference and that CaM isoform selection may contribute to pathway specificity under distinct Ca^2+^ signatures [37]. However, yeast two-hybrid does not establish in planta interaction, Ca^2+^ dependence, or the subcellular site of complex formation. Therefore, in planta validation (BiFC, split-luciferase, or co-immunoprecipitation), together with Ca^2+^ supplementation versus chelation, will be important to test whether the FtIQD19–FtCaM7.2 interaction is Ca^2+^-dependent and where the complex assembles.

Our structural modeling of the Ca^2+^-bound FtIQD19–FtCaM7.2 complex proposes a residue-level interaction mechanism that can be framed in the context of canonical CaM–target recognition. In many CaM–peptide complexes, Ca^2+^-triggered exposure of hydrophobic clefts supports binding to amphipathic helices through a dominant hydrophobic anchor, often complemented by a second hydrophobic position, while surrounding polar contacts provide orientation and stability. In our model, Tyr202 is best interpreted as the primary anchor, whereas nearby hydrophobic residues (e.g., I195 and Y208) remain plausible candidates for secondary anchoring or subsidiary hotspots that complete a two-point clamp. The model also highlights peripheral contacts (salt bridges and hydrogen bonds) that may act as geometric constraints to stabilize a specific binding pose (Figure S3). These inferences remain model-based and should be treated as testable hypotheses. A focused mutational series that separates “anchor” from “reinforcement” residues, combined with quantitative binding assays under Ca^2+^-bound and Ca^2+^-limited conditions, would directly test whether FtIQD19 conforms to a canonical CaM recognition mode.

How might an FtIQD19–CaM module connect to flavonoid-related traits? One plausible route is indirect: Ca^2+^/CaM signaling intersects with stress hormone pathways (including ABA) and transcriptional programs that modulate MYB/bHLH/light regulatory circuits controlling flavonoid biosynthesis [34,36]. A second route is spatial: IQD scaffolds at MTs and/or membrane subdomains could influence vesicle trafficking, organelle positioning, or signaling microdomains that affect enzyme localization or metabolic flux. These scenarios are consistent with published models in which IQDs link Ca^2+^ decoding to cytoskeleton organization and nuclear outputs [10]. Although these mechanisms remain speculative, FtIQD19 is currently the most tractable entry point for testing them, because it is supported not only by association evidence but also by expression-based prioritization, subcellular localization, CaM interaction, and structure-guided hypotheses.

### 3.5. Limitations and Future Perspectives

Several limitations and future directions should be noted. First, although the identified SNPs reached the predefined significance threshold in our *FtIQD*-focused analysis, they have not yet been validated in independent populations or across multiple years and environments. The marked variation in association patterns across altitudinal groups suggests possible genotype-by-environment interactions, but direct measurements of environmental covariates (e.g., temperature, UV radiation, and soil water status) will be required to resolve the underlying drivers [2,3,31]. Because the association patterns differed substantially across altitude strata, further validation in expanded diversity panels and replicated environments will be necessary to distinguish stable genetic effects from context-specific responses [23,32]. The clustered SNP signals near recurrent loci, including *FtIQD22*, *FtIQD16*, *FtIQD19*, and *FtIQD20*, provide a practical basis for marker development (e.g., KASP assays), but these must still be validated in independent populations and breeding materials before routine application [38].

Second, to bridge genotype to biochemical phenotype more directly, future work should integrate stress-treated metabolomics with transcriptomics [25,39]. From the association perspective, loci such as *FtIQD22*, *FtIQD20*, *FtIQD07*, *FtIQD02*, and *FtIQD16* warrant broader follow-up because they represent recurrent or high-signal candidates. However, *FtIQD19* remains the current priority for mechanistic investigation because it combines recurrent association evidence with stress-responsive expression, pathway-linked co-expression, subcellular localization, CaM interaction, and structure-based hypotheses. This makes *FtIQD19* particularly suitable for testing whether perturbation of an *IQD* scaffold can shift flux through flavonol/anthocyanin pathways rather than merely correlating with them.

Third, the FtIQD19–FtCaM7.2 interaction should be validated in planta, together with Ca^2+^ manipulation to test Ca^2+^ dependence, and higher-resolution co-localization to resolve cytoskeletal or membrane-associated contexts [10,11,13]. Finally, stable functional assays, including CRISPR/Cas-mediated knockout, complementation, or transgenic perturbation in tartary buckwheat (or tractable heterologous systems), will be essential to establish causality. Together, these approaches will determine whether *FtIQD19* represents a conserved Ca^2+^/CaM-associated signaling module relevant to stress-responsive metabolism and whether broader *FtIQD* candidates identified here have value for buckwheat improvement.

## 4. Materials and Methods

### 4.1. Identification of IQD Genes in Fagopyrum Tataricum

*IQD* genes in tartary buckwheat (*Fagopyrum tataricum*) were identified using two complementary strategies. First, the genome assembly and annotation were obtained from NCBI (https://www.ncbi.nlm.nih.gov, accessed on 11 May 2024) and the China National Center for Bioinformation (CNCB, https://ngdc.cncb.ac.cn/; assembly accession: GWHBJBL00000000, accessed on 11 May 2024) [40]. The Hidden Markov Model (HMM) profile for the IQ67 domain (Pfam: PF00612) was downloaded from Pfam (http://Pfam.sanger.ac.uk/, accessed on 15 May 2024) [41] and used to scan the buckwheat proteome with HMMER (v3.3) (E-value < 1 × 10^−5^). Second, protein sequences of 33 *Arabidopsis thaliana IQD* genes (*AtIQD*s) were retrieved from TAIR (http://www.arabidopsis.org/, accessed on 14 May 2024) [42] and used as queries to search the buckwheat proteome using BLASTP (E-value < 1 × 10^−10^). Candidate *FtIQD*s were defined as the intersection of HMMER and BLASTP hits, followed by verification of the IQ67 domain using domain annotation tools (see Section 4.6).

### 4.2. Multiple Sequence Alignment and Phylogenetic Analysis

Full-length amino acid sequences of FtIQDs, AtIQDs, and *Oryza sativa* IQDs (OsIQDs) were aligned using MUSCLE implemented in MEGA7 [43,44]. An unrooted phylogenetic tree was constructed in MEGA7 using the neighbor-joining (NJ) method with the p-distance model, 1000 bootstrap replicates, and a 50% bootstrap support cutoff (other parameters default). The tree was visualized using iTOL in a radial layout (https://itol.embl.de/, accessed on 19 June 2024), and FtIQD subfamilies were assigned by reference to the established AtIQD clade framework [9]. An FtIQD-only NJ tree was generated using the same procedure.

### 4.3. Chromosomal Distribution and Synteny Analysis

Chromosomal locations of *FtIQD*s were visualized in TBtools-II (v2.400) using the buckwheat genome annotation [45,46]. Intraspecific collinearity and duplication patterns were analyzed using the One Step MCScanX-Super Fast plugin in TBtools-II [45,46].

For interspecific synteny, protein sequences from six species (*F. tataricum*, *F. dibotrys*, *F. esculentum*, *Beta vulgaris*, *A. thaliana*, and *Glycine max*) were collected [47]. A species phylogeny based on single-copy orthologs was inferred using OrthoFinder2 (v2.5.5) with MAFFT for alignment and IQ-TREE for phylogenetic reconstruction [48,49,50]. Synteny plots were generated using the One Step MCScanX and Dual Synteny Plot plugins in TBtools-II (E-value < 1 × 10^−5^) [46,51]. The order of species in the synteny plot followed the inferred species phylogeny.

### 4.4. Ka/Ks Estimation

Ka/Ks values for duplicated *FtIQD* gene pairs were calculated using the Simple Ka/Ks Calculator (NG method) in TBtools-II (v2.400) [45,46]. CDS sequences for each duplicated pair were extracted, paired according to intraspecific collinearity results, and used to estimate nonsynonymous (Ka) and synonymous (Ks) substitution rates and Ka/Ks ratios to infer selection pressure [52].

### 4.5. Physicochemical Characterization and Prediction of CaM-Binding Segments

Physicochemical properties of FtIQD proteins were calculated using ExPASy ProtParam (https://www.expasy.org) [53], including protein length, molecular weight, and theoretical isoelectric point (pI). Putative CaM-binding segments were predicted using the Calmodulin Target Database (http://calcium.uhnres.utoronto.ca/ctdb/, accessed on 21 June 2024). Predicted CaM-binding segments were compiled and summarized for all FtIQDs.

### 4.6. Motif/Domain Annotation, Gene Structure, and Cis-Element Analysis

Conserved motifs were identified using MEME (v5.5.9) (https://meme-suite.org/meme/, accessed on 23 September 2024), with the number of motifs set to 10 and motif widths allowed to vary from 6 to 60 amino acids [54]. Conserved domains were annotated using the NCBI Batch CD-Search tool (https://www.ncbi.nlm.nih.gov/Structure/bwrpsb/bwrpsb.cgi, accessed on 23 September 2024) [55,56].

For cis-element analysis, 2-kb promoter sequences upstream of each *FtIQD* were extracted using TBtools-II and analyzed using PlantCARE (https://bio.tools/plantcare, accessed on 28 September 2024) [45,46,57]. Cis-elements were manually curated and grouped into seven nonredundant functional categories by merging synonymous annotations: ABA-responsive, DREB-binding, MYB-related, bHLH-binding, AC-type, G-box, and other light-responsive elements [58,59,60,61,62,63]. Gene structure (exon–intron organization) was visualized in TBtools-II using gene models and the FtIQD phylogeny [45,46]. Final composite figures were adjusted for layout and labeling in Adobe Illustrator 2024 [47,64,65].

### 4.7. 3D Structure Prediction of FtIQD Proteins

Protein 3D structures were predicted using the AlphaFold 3 server (https://golgi.sandbox.google.com/, accessed on 15 January 2025) by submitting FtIQD protein sequences [66].

### 4.8. GWAS-Based FtIQD-Focused Association Analysis of Rutin Content

Ten-fold whole-genome resequencing data from tartary buckwheat accessions (PRJNA600676) were used for GWAS [39]. Reads were aligned to the reference genome (assembly accession: GWHBJBL00000000) using BWA (v0.7.17) [35,67]. Variants were called following GATK Best Practices (GATK v4.6.2.0) using per-sample GVCFs and joint genotyping [68,69]. Hard filtering was applied, and only PASS variants were retained. High-quality biallelic SNPs were further filtered using bcftools (v1.19) (MAF > 0.01; site missing rate < 10%) [70]. LD pruning was performed in PLINK 2.0 (window 50 kb, step 5 kb, r^2^ < 0.2), and principal components were used as covariates [71]. From the filtered cohort VCF, variants located within the *FtIQD* gene bodies and their ±5 kb flanking regions were extracted using GATK SelectVariants [72]. To assign each variant to its corresponding gene, we intersected variant coordinates with a BED file of the *FtIQD* intervals using bcftools [70]. Association analysis for rutin traits across five altitude groups, including 1000 m (221 accessions), 1500 m (162 accessions), 2000 m (215 accessions), 2500 m (217 accessions), and 3000 m (171 accessions), was performed under additive, dominant, and recessive genetic models using simple linear regression [39]. Association analysis for quercetin content was also performed across the five altitude groups (see Supplementary Figure S1; Table S4). Because genotype class frequencies differed across altitude groups and across additive, dominant, and recessive encodings, the number of informative observations and analyzable SNPs varied among tests [73]. Because the analysis was restricted to variants within the *FtIQD* interval, a regional significance threshold of *p* < 1 × 10^−5^ (−log_10_(*p*) = 5) was used to define significant loci for downstream interpretation, and a nominal threshold of *p* < 0.05 (−log_10_(*p*) = 1.30) was additionally applied for candidate screening. Manhattan plots were generated using Matplotlib (v3.9.1) [74].

### 4.9. RNA-Seq Analysis of FtIQD Expression

RNA-seq datasets were obtained from NCBI (https://www.ncbi.nlm.nih.gov, accessed on 10 June 2025, BioProject PRJNA522429 for tissue expression) and CNCB (https://ngdc.cncb.ac.cn/, accessed on 10 June 2025; BioProject PRJCA003569 for drought stress) [75]. Reads were aligned to the reference genome using HISAT2 (v2.2.1), and gene-level read counts were obtained using featureCounts (v2.0.6) [76,77]. Genes with very low read counts (counts < 10) were excluded from differential-expression interpretation to reduce spurious signals [78]. Heatmaps were generated using z-score–normalized log_2_(TPM + 1) values.

### 4.10. Correlation Analysis with Flavonol/Anthocyanin Pathway Genes

Spearman correlation coefficients between *FtIQD* expression and flavonol/anthocyanin pathway genes were computed in R using the “psych” package (v2.5.6) [79] and visualized using “corrplot” (v0.95) [80]. Pathway schematics were drawn using FreeChemDraw (https://freechemdraw.com/, accessed on 12 July 2025) and Adobe Illustrator 2024.

### 4.11. Plant Materials and qRT–PCR

Seeds of the tartary buckwheat cultivar ‘GUIMI’ were surface-sterilized, soaked in sterile water for >24 h at room temperature, and germinated on moist filter paper at 30 °C for 3 d. Seedlings were transferred to hydroponic cassettes and grown in a greenhouse (21–23 °C; 16 h light/8 h dark). For independent expression validation, drought stress was simulated by treating 11-day-old seedlings with 20% (*w*/*v*) PEG6000 for 0, 3, 12, and 24 h. Total RNA was extracted using the RNAprep Pure Plant Kit (TIANGEN, Beijing, China) and reverse-transcribed using Evo M-MLV RT Premix (AGbio, Changsha, Hunan, China). qRT–PCR was performed using SYBR Green Premix (AGbio, Changsha, Hunan, China) on an ABI Q5 PCR Real-Time Thermal Cycler (Thermo Fisher Scientific, Wilmington, MA, USA). Expression levels were normalized to *FtH3* and calculated using the 2^−ΔΔCt^ method [81]. Gene-specific primers used for qRT–PCR are listed in Supplementary Table S7.

### 4.12. Subcellular Localization

The *FtIQD19* CDS was cloned into the Gateway vector pMDC83 to generate a 35S-driven FtIQD19–GFP fusion [82]. Constructs were introduced into *Agrobacterium tumefaciens* GV3101 and infiltrated into fully expanded leaves of 6-week-old *Nicotiana benthamiana* (22 °C; 16 h light/8 h dark) [83]. At 48 h post-infiltration, nuclei were counterstained with DAPI, and images were captured using a confocal microscope (STELLARIS 5, Leica, Wetzlar, Germany). Primers used for cloning FtIQD19 into pMDC83 are provided in Supplementary Table S8.

### 4.13. Prediction of FtIQD19 Interaction Network with FtCaMs

Putative FtCaM proteins were identified by BLASTP against the buckwheat proteome using Arabidopsis CaM sequences as queries (E-value < 1 × 10^−10^). Structural complexes between FtIQD19 and each FtCaM were predicted using AlphaFold Multimer via the AlphaFold Server (https://alphafoldserver.com, accessed on 20 October 2025), and interaction confidence was summarized using the interface-predicted TM-score (ipTM) [66,84]. For network visualization, ipTM values were min–max normalized to obtain ipTM_normalized (0.1–1). The FtIQD19–FtCaM interaction network was visualized in Cytoscape (v3.10.4), with edge length and transparency inversely scaled to ipTM_normalized; shorter and darker edges (lower transparency) indicate stronger predicted interactions [85].

### 4.14. Yeast Two-Hybrid Assays

Yeast two-hybrid (Y2H) assays were performed to validate interactions between FtIQD19 and selected FtCaM proteins [86]. Full-length FtIQD19 was cloned into pGBKT7 (BD) (Takara, Beijing, China), and FtCaM7.2, FtCaM7.4, FtCaM7.5, and FtCaM7.6 were cloned into pGADT7 (AD) (Takara, Beijing, China). Constructs were co-transformed into the yeast strain Y2HGold. Transformants were selected on SD/−Leu/−Trp (SD/−WL) and screened on SD/−Leu/−Trp/−His/−Ade (SD/−WLHA) supplemented with X-α-Gal. Empty vectors and a known interacting pair were included as negative and positive controls, respectively. Primers used for constructing Y2H vectors (pGBKT7-FtIQD19 and pGADT7-FtCaM7.2/7.4/7.5/7.6) are provided in Supplementary Table S8.

### 4.15. Structural Modeling of the FtIQD19–FtCaM7.2 Interaction

Sequences of FtCaM7.2 and FtIQD19 were submitted to the AlphaFold Server (https://alphafoldserver.com, accessed on 10 December 2025) to predict FtCaM7.2 alone, Ca^2+^-bound FtCaM7.2 (four Ca^2+^ ions), FtIQD19 alone, and the Ca^2+^-bound FtCaM7.2–FtIQD19 complex [66,84]. The highest-confidence complex model was visualized in PyMOL (v3.1.6.1) [87]. Atom pairs with distances ≤3.5 Å were screened as candidate contacts. Key interface residues were summarized based on residue properties and known CaM–target interaction features.

## 5. Conclusions

This study provides a genome-wide resource for the IQ67-domain (IQD) gene family in tartary buckwheat (*Fagopyrum tataricum*), identifying 24 *FtIQD*s and describing their evolutionary features, promoter architectures, and divergent expression profiles. By integrating FtIQD-focused association signals across altitudinal environments with stress-responsive expression and pathway co-expression analyses, we prioritized *FtIQD19* as a candidate for follow-up studies on rutin-related traits. FtIQD19 shows predominant nuclear localization with additional filamentous/peripheral signals and interacts with FtCaM7.2 in yeast two-hybrid assays, while structural modeling of the Ca^2+^-bound FtIQD19–FtCaM7.2 complex suggests testable residue-level interaction hypotheses. Collectively, these findings establish a foundation for dissecting Ca^2+^/CaM–IQD modules potentially involved in environment-dependent rutin variation and stress adaptation in tartary buckwheat.

## Figures and Tables

**Table 1:** Physicochemical properties of the 24 FtIQD proteins identified in tartary buckwheat.

Gene Name	Gene ID	Chr	Genomic Length (bp)	CDS Length (bp)	Protein		
					Length (aa)	Mw (Da)	pI
*FtIQD01*	GWHGBJBL000824	1	2051	1233	410	47,169.33	10.10
*FtIQD02*	GWHGBJBL001343	1	1771	1020	339	39,479.56	10.50
*FtIQD03*	GWHGBJBL003199	2	1845	1254	417	47,042.69	9.99
*FtIQD04*	GWHGBJBL003336	2	2849	1350	449	49,314.72	10.54
*FtIQD05*	GWHGBJBL003633	2	1350	1086	361	40,750.72	10.41
*FtIQD06*	GWHGBJBL004162	2	1150	900	299	33,382.81	10.35
*FtIQD07*	GWHGBJBL006577	2	3829	1293	430	48,686.38	9.93
*FtIQD08*	GWHGBJBL008040	2	1592	1206	401	45,837.43	10.11
*FtIQD09*	GWHGBJBL009888	3	1774	1257	418	46,487.75	10.56
*FtIQD10*	GWHGBJBL009970	3	1742	1425	474	51,742.72	10.06
*FtIQD11*	GWHGBJBL010283	3	2825	1233	410	46,006.21	10.26
*FtIQD12*	GWHGBJBL015827	4	1891	1290	429	47,657.10	10.13
*FtIQD13*	GWHGBJBL016226	4	3224	1467	488	53,554.38	9.83
*FtIQD14*	GWHGBJBL017979	4	3418	1287	428	48,281.93	10.12
*FtIQD15*	GWHGBJBL019563	5	2537	1212	403	45,714.11	10.25
*FtIQD16*	GWHGBJBL019830	5	2505	1536	511	57,676.82	10.28
*FtIQD17*	GWHGBJBL020415	5	1568	1308	435	49,215.42	9.49
*FtIQD18*	GWHGBJBL024096	6	877	789	262	28,716.25	10.12
*FtIQD19*	GWHGBJBL025678	7	2916	1641	546	61,736.02	10.32
*FtIQD20*	GWHGBJBL025945	7	1542	1140	379	42,337.35	10.22
*FtIQD21*	GWHGBJBL029147	7	1658	1257	418	47,081.47	9.91
*FtIQD22*	GWHGBJBL029333	8	1119	1035	344	38,684.76	10.10
*FtIQD23*	GWHGBJBL030137	8	623	531	176	19,915.80	9.76
*FtIQD24*	GWHGBJBL030820	8	2984	1386	461	51,532.97	9.55

## Data Availability

The data presented in this study are available in the China National Center for Bioinformation at https://ngdc.cncb.ac.cn/search/all?&q=GWHBJBL00000000 and https://ngdc.cncb.ac.cn/bioproject/browse/insdc/PRJNA600676, accessed on 11 May 2024.

## Supplementary Materials

The following supporting information can be downloaded at: https://www.mdpi.com/article/10.3390/plants15081212/s1. Supplementary Figure S1: *FtIQD*-focused association analysis of quercetin traits across five altitudes (3000 m, 2500 m, 2000 m, 1500 m, and 1000 m), showing –log10(*p*) values for SNPs within *FtIQD* loci under additive, dominant, and recessive models. Supplementary Figure S2: Spearman correlation heatmap showing the relationships between *FtIQD* gene expression and key genes in the flavonoid/anthocyanin biosynthesis pathway. Supplementary Figure S3: Structural model highlighting ancillary interactions that stabilize the primary anchoring interface in the FtIQD19–FtCaM7.2 complex. Supplementary Table S1: Predicted calmodulin-binding sites identified in the 24 FtIQD proteins. Supplementary Table S2: Ka/Ks values and estimated divergence times for duplicated *FtIQD* gene pairs. Supplementary Table S3: List of significant SNP sites identified in rutin association analysis across different altitudes within *FtIQD* loci. Supplementary Table S4: List of significant SNP sites identified in quercetin association analysis across different altitudes within *FtIQD* loci. Supplementary Table S5: Results of ANOVA analysis for qRT-PCR experiments. Supplementary Table S6: *FtIQD* and *FtCaM* gene list with corresponding original gene IDs identified by BLAST. Supplementary Table S7: Primers used for qRT-PCR validation. Supplementary Table S8: Primers used for subcellular localization and yeast two-hybrid assays.
