# Supplementary material for: A Ca2+/Calmodulin-Interacting IQD Hub in Tartary Buckwheat: Genome-Wide FtIQD Analysis and Characterization of FtIQD19
Source: Plants (Basel). 2026 Apr 15;15(8):1212. doi: 10.3390/plants15081212 (PMC13119371; doi:10.3390/plants15081212)
Supplement: Supplementary file 1 [file plants-15-01212-s001.zip › plants-4221141-supplementary figures.pdf]

Supplementary Figures

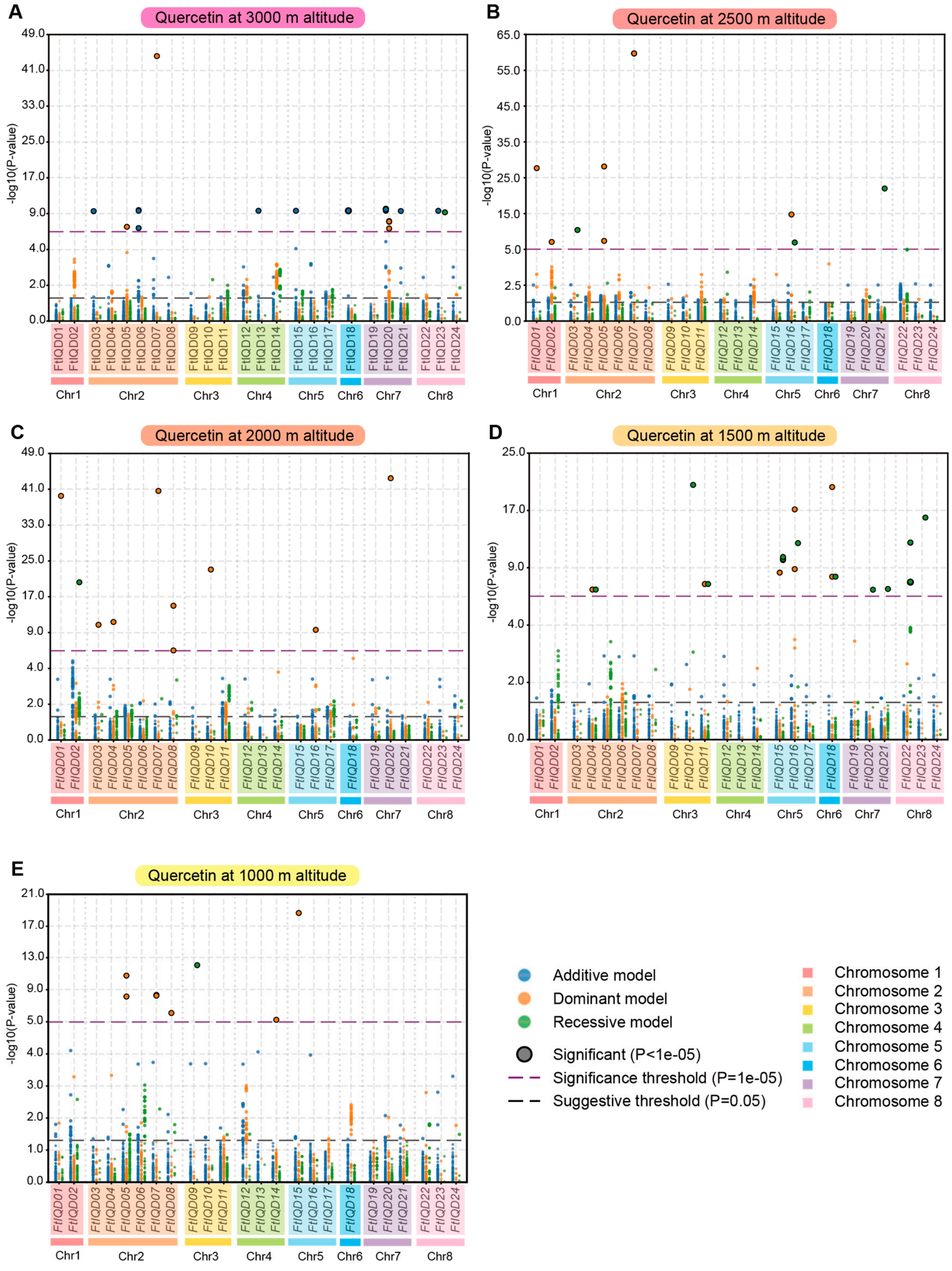

Figure S1.

FtIQD-focused association analysis of quercetin traits across altitudes of 3000m (A), 2500m (B), 2000m (C), 1500m (D) and 1000m (E). The x-axis shows candidate *IQD* genes, and the y-axis shows association significance as $-\log_{10}(P)$. The same color block denotes the same chromosome. For each

gene, results from additive, dominant, and recessive models are presented simultaneously. The 24 *FtIQD*s are ordered by chromosomal positions from chromosomes 1 to 8. Column colors indicate chromosomes. The red dashed line denotes the suggestive threshold (P = 0.05; $-\log_{10}(P) = 1.30$), and the purple dashed line indicates the conventional GWAS significance threshold ($-\log_{10}(P) = 5$). Red bold dots with black borders indicate the most significant SNPs in each panel.

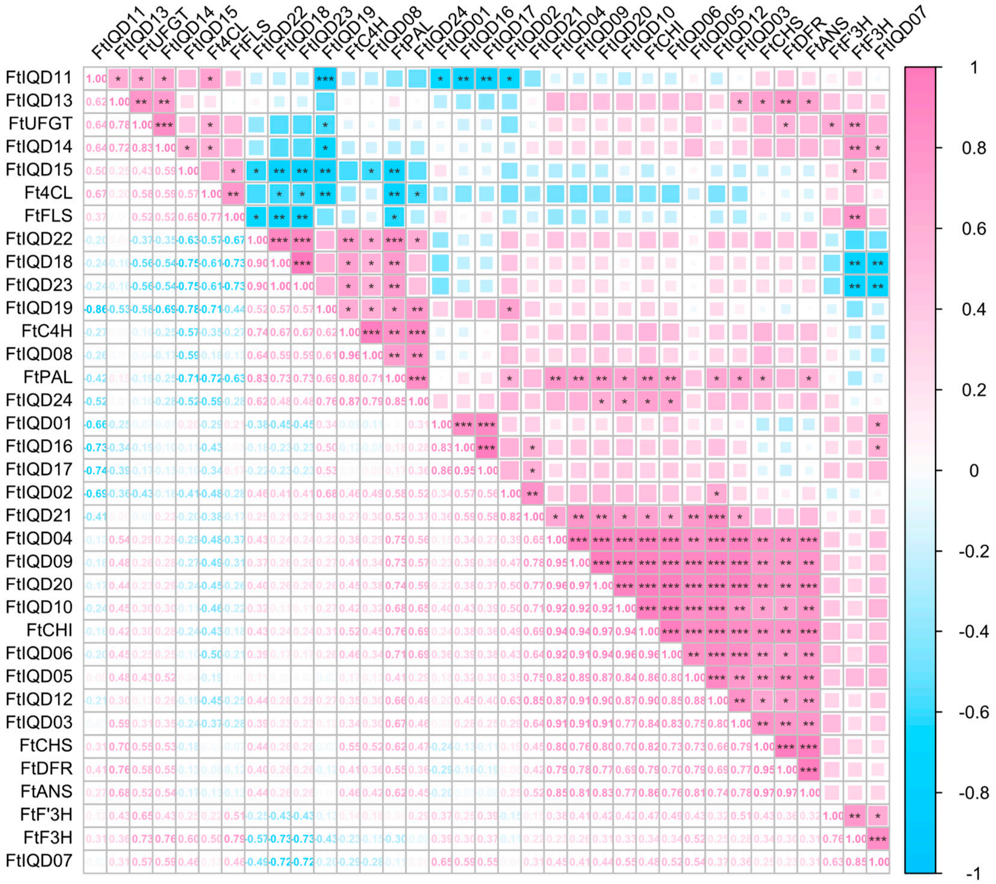

Figure S2.

Spearman correlation heatmap of *FtIQD* genes with key genes in the flavonoid/anthocyanin biosynthesis pathway. The heatmap showed Spearman correlation coefficients between *FtIQD* family genes and key genes in the flavonoid/anthocyanin biosynthesis pathway. The color gradient represented the strength of correlation (pink for positive correlation, blue for negative correlation, with color intensity corresponding to the absolute value of the coefficient). Asterisks indicated significance levels (*P<0.05, **P<0.01**,** ***P<0.001).

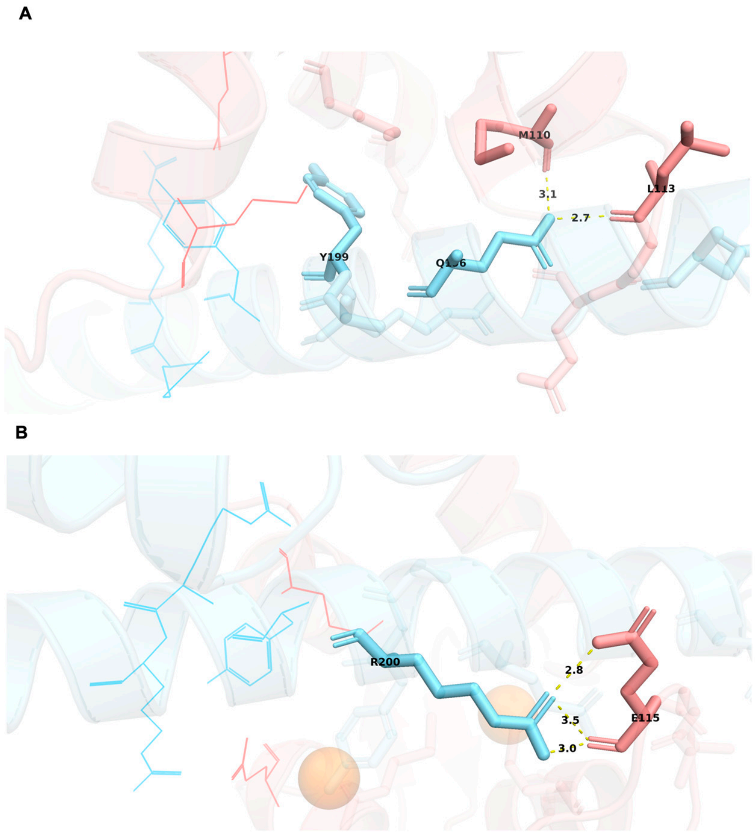

Figure S3.

Ancillary interactions stabilizing the primary anchoring interface. (A) Hydrophobic patch reduces water access near the anchor (B) Salt-bridge belt cinches the binding peptide. Note that the aromatic anchor-and-pocket interactions (Results 2.6) were shown as lines, whereas these ancillary interactions were displayed as sticks.
