# Supplementary material for: A Ca2+/Calmodulin-Interacting IQD Hub in Tartary Buckwheat: Genome-Wide FtIQD Analysis and Characterization of FtIQD19
Source: Plants (Basel). 2026 Apr 15;15(8):1212. doi: 10.3390/plants15081212 (PMC13119371; doi:10.3390/plants15081212)
Supplement: Supplementary file 1 [file plants-15-01212-s001.zip › plants-4221141-supplementary tables.pdf]

Supplementary Tables

Table S1. List of predicted calmodulin-binding sites in 24 FtIQDs proteins

Protein name	Predicted calmodulin binding sequence	
FtIQD01	225-I**LLSKTGAMLRRHRIKA**Y	
FtIQD02	125-RLQ**AIVRGRAVRRQ**VSN	
FtIQD03	209-LNREKALAYAFSQQLWRF	
FtIQD04	186-KWAAIAIQSHFRGY	184-IQ**AMLRAQAR**AR
FtIQD05	97-TT**HHSRFIVAGRE**NLA	143-RGYL**VRKQTSSTL**Y
FtIQD06	83-PKNFLIVRKEW**AAIRIQ**T	
FtIQD07	5-G**KWIKALVGLKKSEKS**H	
FtIQD08	357-N**ALLKSGSYRQRSYTGFK**	
FtIQD09	125-VRGRQ**VRKQAAVTLRCMQ**ALVRVQARVRAR	
FtIQD10	177-QAL**IRAQATIRSKR**VI	
FtIQD11	142-TTVG**RSRARFA**ALK	
FtIQD12	179-QAS**ARAQRARR**SIG	
FtIQD13	149-G**LVKLQALVRGHIMRKKT**A	
FtIQD14	5-R**KWIKALVGFKKTDKS**Q	
FtIQD15	68-AE**VVRLTSRRGGDISL**RR	
FtIQD16	137-LV**KLQALVRGHN**VR	
FtIQD17	6-K**WIKSFLVGGKKDKE**K	167-RCMQALVTAQARARA
FtIQD18	193-E**KAIKIQAAFRGHLARRAF**	
FtIQD19	1-MGKKGSWFSAI	
FtIQD20	85-PKDFLVIRQEW**AAIRIQ**T	
FtIQD21	162-T**LRCMQALLTAQARARA**QR	
FtIQD22	88-TI**QTNFRGYLARK**AL	115-VR**GYLVRKQAN**AT
FtIQD23	89-RID**AAVRIQSFFRGHLA**R	
FtIQD24	108-YSRH**SKEE**KAAV	

Notes: Putative calmodulin-binding sites were predicted using the Calmodulin Target Database. Only amino acid segments with prediction scores ≥ 7 were shown, and residues with the highest score (9) are highlighted in bold.

Table S2. Ka/Ks calculation and estimated divergence time for the duplicated *FtIQD* gene pairs

No.	Paralogous Pair	Ka	Ks	Ka/Ks	Divergence time (MYA)
1	*FtIQD05-FtIQD11*	0.4575	3.3942	0.1378	113.1391
2	*FtIQD04-FtIQD13*	0.2642	1.4209	0.1859	47.3643
3	*FtIQD07-FtIQD14*	0.2118	0.5894	0.3593	19.6452
4	*FtIQD11-FtIQD12*	0.4019	2.3279	0.1726	77.5952
5	*FtIQD17-FtIQD21*	0.1685	0.9193	0.1833	30.6444

Table S3. Significant sites in rutin association analysis across altitudes at *FtIQD* loci

Elevation	Gene	Chromosome	Position	Ref	Alt	Model	P_value
1000	*FtIQD01*	Chr1	12725217	G	A	additive	1.541642e-06
1000	*FtIQD02*	Chr1	25466182	C	G	dominant	1.257081e-06
1000	*FtIQD04*	Chr2	4803209	T	C	dominant	2.847753e-14
1000	*FtIQD16*	Chr5	22990182	G	A	additive	1.413090e-06
1000	*FtIQD19*	Chr7	9891424	C	T	dominant	5.208792e-07
1000	*FtIQD20*	Chr7	13903173	C	T	dominant	2.190352e-31
1000	*FtIQD20*	Chr7	13894983	G	A	dominant	1.654160e-06
1000	*FtIQD21*	Chr7	51735643	A	C	additive	1.225649e-06
1000	*FtIQD23*	Chr8	14227212	C	T	additive	1.309634e-06
1000	*FtIQD23*	Chr8	14229750	T	C	additive	1.467801e-06
1500	*FtIQD11*	Chr3	15474738	C	T	dominant	8.853472e-07
1500	*FtIQD11*	Chr3	15474738	C	T	recessive	8.853472e-07
1500	*FtIQD21*	Chr7	51737207	A	T	dominant	5.256647e-07
1500	*FtIQD22*	Chr8	1693698	G	A	dominant	1.382796e-15
1500	*FtIQD22*	Chr8	1697823	C	G	recessive	1.997320e-13
1500	*FtIQD22*	Chr8	1698958	T	C	recessive	2.679310e-13
1500	*FtIQD22*	Chr8	1697553	G	A	recessive	3.297143e-13
1500	*FtIQD22*	Chr8	1692242	T	G	recessive	4.561117e-13
1500	*FtIQD22*	Chr8	1696050	T	C	recessive	4.561117e-13
1500	*FtIQD22*	Chr8	1692070	T	A	recessive	6.186251e-13
1500	*FtIQD22*	Chr8	1691880	G	A	recessive	7.349948e-13
1500	*FtIQD22*	Chr8	1691417	A	G	recessive	8.224770e-13
1500	*FtIQD22*	Chr8	1693008	G	C	recessive	8.366127e-13
1500	*FtIQD22*	Chr8	1693332	A	G	recessive	8.993397e-13
1500	*FtIQD22*	Chr8	1693827	T	C	recessive	1.003011e-12
2000	*FtIQD02*	Chr1	25465917	G	A	additive	7.022107e-17
2000	*FtIQD02*	Chr1	25467472	C	T	recessive	3.655564e-10
2000	*FtIQD03*	Chr2	3469397	G	A	additive	1.360803e-16
2000	*FtIQD04*	Chr2	4806090	G	A	additive	1.232503e-16
2000	*FtIQD05*	Chr2	7873776	C	T	dominant	6.934008e-10
2000	*FtIQD06*	Chr2	15281299	C	T	additive	1.043360e-16
2000	*FtIQD07*	Chr2	45748734	G	A	recessive	8.037985e-29
2000	*FtIQD13*	Chr4	35272068	G	C	recessive	4.520402e-07
2000	*FtIQD15*	Chr5	15606352	A	G	additive	1.447419e-16
2000	*FtIQD16*	Chr5	22991474	C	T	additive	1.662489e-16
2000	*FtIQD16*	Chr5	22990422	G	A	additive	1.149546e-11
2000	*FtIQD16*	Chr5	22997448	C	G	dominant	4.137006e-11
2000	*FtIQD16*	Chr5	22997448	C	G	recessive	4.137006e-11
2000	*FtIQD17*	Chr5	41112376	T	C	dominant	3.807500e-06

Elevation	Gene	Chromosome	Position	Ref	Alt	Model	P_value
2000	*FtIQD19*	Chr7	9890306	A	C	dominant	1.961651e-11
2000	*FtIQD19*	Chr7	9890306	A	C	recessive	1.961651e-11
2500	*FtIQD02*	Chr1	25475386	G	C	recessive	1.756183e-06
2500	*FtIQD02*	Chr1	25465579	A	T	recessive	6.846609e-06
2500	*FtIQD02*	Chr1	25465955	T	C	recessive	6.893048e-06
2500	*FtIQD12*	Chr4	30325551	T	A	dominant	7.501726e-06
2500	*FtIQD19*	Chr7	9889289	G	A	recessive	9.534533e-07
2500	*FtIQD20*	Chr7	13905930	C	T	dominant	3.246750e-06
2500	*FtIQD22*	Chr8	1698019	C	T	dominant	2.095817e-11
3000	*FtIQD05*	Chr2	7873776	C	T	dominant	2.273293e-13
3000	*FtIQD10*	Chr3	12329630	A	T	dominant	1.209571e-08

Table S4. Significant sites in quercetin association analysis across altitudes at *FtIQD* loci

Elevation	Gene	Chromosome	Position	Ref	Alt	Model	P_value
1000	FtIQD05	Chr2	7873776	C	T	dominant	1.727964e-11
1000	FtIQD05	Chr2	7871180	G	A	dominant	7.020489e-09
1000	FtIQD07	Chr2	45751115	C	T	dominant	4.542422e-09
1000	FtIQD07	Chr2	45751119	A	T	dominant	4.542422e-09
1000	FtIQD07	Chr2	45751233	C	T	dominant	5.441191e-09
1000	FtIQD08	Chr2	57851939	T	A	dominant	7.950938e-07
1000	FtIQD09	Chr3	11684126	C	T	recessive	8.560719e-13
1000	FtIQD14	Chr4	52420327	T	G	dominant	5.632385e-06
1000	FtIQD15	Chr5	15608252	A	C	dominant	2.181729e-19
1500	FtIQD04	Chr2	4804807	C	T	dominant	1.102323e-06
1500	FtIQD04	Chr2	4804807	C	T	recessive	1.102323e-06
1500	FtIQD10	Chr3	12330043	A	G	recessive	2.747143e-21
1500	FtIQD11	Chr3	15476220	C	T	dominant	1.858536e-07
1500	FtIQD11	Chr3	15476220	C	T	recessive	1.858536e-07
1500	FtIQD15	Chr5	15610788	A	G	recessive	3.127967e-11
1500	FtIQD15	Chr5	15608345	G	T	recessive	5.745207e-11
1500	FtIQD15	Chr5	15609245	T	G	recessive	6.209130e-11
1500	FtIQD15	Chr5	15608375	A	G	recessive	8.513911e-11
1500	FtIQD15	Chr5	15605817	C	T	dominant	4.779650e-09
1500	FtIQD16	Chr5	22991376	G	A	dominant	7.116188e-18
1500	FtIQD16	Chr5	22991376	G	A	recessive	3.682320e-13
1500	FtIQD16	Chr5	22988770	C	T	dominant	1.540591e-09
1500	FtIQD18	Chr6	39758813	G	A	dominant	5.470154e-21
1500	FtIQD18	Chr6	39759509	A	G	dominant	1.758300e-08
1500	FtIQD18	Chr6	39759509	A	G	recessive	1.758300e-08

Elevation	Gene	Chromosome	Position	Ref	Alt	Model	P_value
1500	FtIQD20	Chr7	13895280	G	C	recessive	1.160635e-06
1500	FtIQD21	Chr7	51737606	C	T	recessive	9.162750e-07
1500	FtIQD22	Chr8	1698051	G	A	recessive	2.962530e-13
1500	FtIQD22	Chr8	1695267	G	A	recessive	3.201088e-13
1500	FtIQD22	Chr8	1701363	A	C	recessive	9.211358e-08
1500	FtIQD22	Chr8	1693574	C	G	recessive	1.029765e-07
1500	FtIQD22	Chr8	1695347	A	G	recessive	1.093948e-07
1500	FtIQD22	Chr8	1695283	A	G	recessive	1.094642e-07
1500	FtIQD22	Chr8	1695500	A	G	recessive	1.107894e-07
1500	FtIQD23	Chr8	14228989	G	A	recessive	9.947867e-17
2000	FtIQD01	Chr1	12719195	G	A	dominant	3.015112e-40
2000	FtIQD02	Chr1	25465656	C	T	recessive	5.827122e-21
2000	FtIQD03	Chr2	3471209	G	T	dominant	1.822297e-11
2000	FtIQD04	Chr2	4803209	T	C	dominant	4.273880e-12
2000	FtIQD07	Chr2	45749249	A	T	dominant	2.315666e-41
2000	FtIQD08	Chr2	57850443	A	G	dominant	1.013020e-15
2000	FtIQD08	Chr2	57849533	C	T	dominant	9.621126e-06
2000	FtIQD10	Chr3	12327528	C	T	dominant	8.333904e-24
2000	FtIQD16	Chr5	22999288	G	C	dominant	2.460248e-10
2000	FtIQD20	Chr7	13895349	G	A	dominant	3.388361e-44
2500	FtIQD01	Chr1	12719005	C	T	dominant	2.092239e-28
2500	FtIQD02	Chr1	25465656	C	T	dominant	8.161839e-08
2500	FtIQD03	Chr2	3466321	A	G	recessive	3.821782e-11
2500	FtIQD05	Chr2	7873776	C	T	dominant	7.033507e-29
2500	FtIQD05	Chr2	7867705	C	T	dominant	4.146754e-08
2500	FtIQD07	Chr2	45751233	C	T	dominant	1.951544e-60
2500	FtIQD07	Chr2	45751115	C	T	dominant	1.981604e-60
2500	FtIQD07	Chr2	45751119	A	T	dominant	1.981604e-60
2500	FtIQD16	Chr5	22989913	G	A	dominant	1.775696e-15
2500	FtIQD16	Chr5	22991376	G	A	recessive	1.169710e-07
2500	FtIQD21	Chr7	51737573	C	T	recessive	9.899210e-23
3000	FtIQD03	Chr2	3473879	G	A	additive	2.804426e-10
3000	FtIQD05	Chr2	7867705	C	T	dominant	8.796665e-07
3000	FtIQD06	Chr2	15272166	T	C	additive	1.622505e-10
3000	FtIQD06	Chr2	15272314	T	C	additive	2.276825e-10
3000	FtIQD06	Chr2	15275336	G	T	additive	1.662821e-06
3000	FtIQD07	Chr2	45741842	C	T	dominant	6.622418e-45
3000	FtIQD13	Chr4	35274239	G	T	additive	2.276825e-10
3000	FtIQD15	Chr5	15609061	A	G	additive	2.276825e-10
3000	FtIQD18	Chr6	39760770	C	T	additive	2.276825e-10

Elevation	Gene	Chromosome	Position	Ref	Alt	Model	P_value
3000	FtIQD18	Chr6	39757132	G	A	additive	2.283651e-10
3000	FtIQD20	Chr7	13903077	G	A	additive	7.947427e-11
3000	FtIQD20	Chr7	13903105	A	C	additive	8.362911e-11
3000	FtIQD20	Chr7	13903082	A	T	additive	8.849972e-11
3000	FtIQD20	Chr7	13903075	G	T	additive	9.045657e-11
3000	FtIQD20	Chr7	13903110	A	T	additive	1.079188e-10
3000	FtIQD20	Chr7	13902992	A	G	additive	2.244083e-10
3000	FtIQD20	Chr7	13896391	C	T	dominant	4.595793e-08
3000	FtIQD20	Chr7	13896434	C	A	dominant	5.715709e-08
3000	FtIQD20	Chr7	13896416	G	A	dominant	6.610137e-08
3000	FtIQD20	Chr7	13894983	G	A	dominant	2.086332e-06
3000	FtIQD21	Chr7	51738720	C	T	additive	2.570357e-10
3000	FtIQD23	Chr8	14234052	C	T	additive	2.336904e-10
3000	FtIQD23	Chr8	14230241	A	G	recessive	5.093297e-10

Table S5. ANOVA test of qRT-PCR result

Gene Name	Drought Stress (Significance)
FtIQD01	0.0669 (.)
FtIQD04	0.000378 (***)
FtIQD07	0.000205 (***)
FtIQD19	0.00258 (**)
FtIQD21	0.0669 (.)
FtIQD24	6.91e-05 (***)

Significance codes: . P > 0.05; * P < 0.05; ** P < 0.01; *** P < 0.001;

Table S6. *FtIQD* and *FtCaM* Gene List and Corresponding Original Gene IDs from BLAST

Gene Type	Gene Name	Original Gene ID
	FtIQD01	*GWHGBJBL000824*
	FtIQD02	*GWHGBJBL001343*
	FtIQD03	*GWHGBJBL003199*
	FtIQD04	*GWHGBJBL003336*
FtIQD genes	*FtIQD05*	*GWHGBJBL003633*
	FtIQD06	*GWHGBJBL004162*
	FtIQD07	*GWHGBJBL006577*
	FtIQD08	*GWHGBJBL008040*
	FtIQD09	*GWHGBJBL009888*

Gene Type	Gene Name	Original Gene ID
	FtIQD10	*GWHGBJBL009970*
	FtIQD11	*GWHGBJBL010283*
	FtIQD12	*GWHGBJBL015827*
	FtIQD13	*GWHGBJBL016226*
	FtIQD14	*GWHGBJBL017979*
	FtIQD15	*GWHGBJBL019563*
	FtIQD16	*GWHGBJBL019830*
	FtIQD17	*GWHGBJBL020415*
	FtIQD18	*GWHGBJBL024096*
	FtIQD19	*GWHGBJBL025678*
	FtIQD20	*GWHGBJBL025945*
	FtIQD21	*GWHGBJBL029147*
	FtIQD22	*GWHGBJBL029333*
	FtIQD23	*GWHGBJBL030137*
	FtIQD24	*GWHGBJBL030820*
FtCaM genes	*FtCaM7.1*	*GWHGBJBL009808*
	FtCaM7.2	*GWHGBJBL013691*
	FtCaM6.1	*GWHGBJBL018601*
	FtCaM7.3	*GWHGBJBL019626*
	FtCaM7.4	*GWHGBJBL020078*
	FtCaM8.1	*GWHGBJBL020424*
	FtCaM3.1	*GWHGBJBL021834*
	FtCaM7.5	*GWHGBJBL027244*
	FtCaM7.6	*GWHGBJBL029443*
	FtCaM7.7	*GWHGBJBL032346*

Table S7. Primers in qRT-PCR

Gene Name	Forward Primer (5`-3`)	Reverse Primer (5`-3`)	Amplicon size (bp)
FtIQD01	CAACATACATGGCTCCAAC	TTCCCACTAATAACACTACCC	184
FtIQD04	GTTCAAGGTTCTTCTCCAGG	GAATCGACGGTAAATTAGCA	163
FtIQD07	TGTACGACCTTGGGAGAA	CTGACTAGCCTGGTTTGG	169
FtIQD19	ATAGCCTTACGAGTTGCC	ATGAGCCTCTCTTATTCCAC	168
FtIQD21	AGAAAGGAATGATGGAGGAG	CTGATGGTGCTGGTGAGA	195
FtIQD24	GCTGTCCTCGTTCAATCTTA	CTCTGGCTTGGACTCTTACC	166

Table S8. Primers in subcellular localization and Yeast two-hybrid

Assays	Gene Name	Primer name	Primer Sequence (5`-3`)
Subcellular localization	*FtIQD19*	FtIQD19-F	ATGGGGAAGAAAGGAAGCTGGT
		FtIQD19-R	CACAAATCTGTTAAATGGCCTCCTT
Yeast two-hybrid	*FtIQD19*	BDFtIQD19-F	CATGGAGGCCGAATTCATGGGGAAGAAAGGAAGCTG
		BDFtIQD19-R	GCAGGTCGACGGATCCCTCACACAAATCTGTTAAATGGCCTC
	FtCaM7.4	ADFtCaM7.4-F	GGAGGCCAGTGAATTCATGGCGGAACAACTCACC
		ADFtCaM7.4-R	CGAGCTCGATGGATCCCTCACTTGGCCATCATGACC
	FtCaM8.1	ADFtCaM8.1-F	GGAGGCCAGTGAATTCATGGCTATGGCAGAAGCTG
		ADFtCaM8.1-R	CGAGCTCGATGGATCCCTCATAGTGACGTCATGAATTTGACAAAG
	FtCaM3,1	ADFtCaM3.1-F	GGAGGCCAGTGAATTCATGGCAGACCAGTTGACC
		ADFtCaM3.1-R	CGAGCTCGATGGATCCCCTAAAGGATTACACACTTATCCCTTCTT
	FtCaM7.7	ADFtCaM7.7-F	GGAGGCCAGTGAATTCATGGCTGATCAACTCACCG
		ADFtCaM7.7-R	CGAGCTCGATGGATCCCTTACTTGGCCATCATCACCTTAAC
	FtCaM7.2	ADFtCaM7.2-F	GGAGGCCAGTGAATTCATGGCAGACCAGTTGACC
		ADFtCaM7.2-R	CGAGCTCGATGGATCCCCTAAAGATAAAAAAATTTGGCCATCATA
	FtCaM7.5	ADFtCaM7.5-F	GGAGGCCAGTGAATTCATGGCGGACCAACTCACC
		ADFtCaM7.5-R	CGAGCTCGATGGATCCCTTACTTGGCCATCATAACCTTGAC
	FtCaM6.1	ADFtCaM6.1-F	GGAGGCCAGTGAATTCATGGCCGACCAGTTGACA
		ADFtCaM6.1-R	CGAGCTCGATGGATCCCTTAGAGAATTGGACACCAACTCTTC
	FtCaM7.6	ADFtCaM7.6-F	GGAGGCCAGTGAATTCATGGCCGACCAGTTGACC
		ADFtCaM7.6-R	CGAGCTCGATGGATCCCTCACTTGGCCATCATAACCTTGAC
	FtCaM7.1	ADFtCaM7.1-F	GGAGGCCAGTGAATTCATGGCCGATCAGTTGACC
		ADFtCaM7.1-R	CGAGCTCGATGGATCCCTCACTTGGCCATCATGACC
